# Endoscopic Treatment of a Severe Vaginal Stenosis Following Battery Insertion in an 11-Year-Old Girl

**DOI:** 10.1055/a-1920-5849

**Published:** 2022-09-19

**Authors:** Riccardo Guanà, Andrea Carpino, Giuseppe Garbagni, Cecilia Morchio, Salvatore Garofalo, Alessandro Pane, Federico Scottoni, Elisa Zambaiti, Giulia Perucca, Elena Madonia, Fabrizio Gennari

**Affiliations:** 1 Division of Pediatric Surgery, Regina Margherita Children's Hospital, Turin, Italy; 2 Department of Pediatrics, Ospedale Infantile Regina Margherita, Turin, Piemonte, Italy; 3 Department of Pediatric Gynaecology, Regina Margherita Children's Hospital, Turin, Piemonte, Italy; 4 Department of Pediatric Surgery, Ospedale Infantile Regina Margherita, Turin, Piemonte, Italy; 5 Department of Surgery and Transplantation Centre, Bambino Gesù Children's Hospital - Bambino Gesù Children's Hospital, Rome, Italy; 6 University College London Institute of Child Health, London, United Kingdom; 7 Department of Women's and Children's Health, Pediatric Surgery, Universita degli Studi di Padova Dipartimento di Medicina, Padova, Italy; 8 Pediatric Radiology Unit, Regina Margherita Children's Hospital, Turin, Italy

**Keywords:** children, endoscopic treatment, hematometrocolpos, vaginal stenosis

## Abstract

Acquired vaginal strictures are rare entities in children. As a result, they are generally difficult to manage and tend to recur despite appropriate initial therapy. This case study reports the staged management of vaginal stenosis following the insertion of a button battery. In this case, an 11-year-old girl experienced at 4 years old a battery insertion in the vaginal canal by her neighbor's son, who was 6-year-old at the time. Two weeks from insertion, the parents noted the foreign body discharge spontaneously. The girl had not complained of any symptoms at the time and had been asymptomatic for many years. In November 2020, she came to the emergency department reporting cramping abdominal pain accompanied by mucopurulent discharge. An abdominal ultrasound showed the presence of hematometrocolpos, and a vaginal stenosis dilation under general anesthesia was performed the following day. After 3 weeks, the stenosis was still present, preventing the passage of Hegar number 4. The girl was subjected to a vaginoscopic stenosis resection utilizing a monopolar hook passed through an operative channel. A Bakri catheter filled with 120 mL of water was left in place. After 10 days, the girl was discharged home with the Bakri inserted. Two weeks after discharge, she was reevaluated in the outpatient setting, where the Bakri was removed with no signs of residual stenosis. Acquired vaginal stenosis could be demanding to treat, particularly with the sole conservative approach. A first-line option can be the Hegar dilation. The endoscopic approach can be a second-line, minimally invasive treatment, but long-term outcomes are difficult to predict.

## Introduction

Acquired vaginal strictures are rare entities in children, often being iatrogenic, postsurgical, or postforeign body introduction. Acquired vaginal strictures, which occur after the introduction of a foreign body, are generally difficult to manage and tend to recur despite appropriate initial therapy. Congenital strictures are equally rare and are most represented by the transverse vaginal septum (TVS).

The diagnosis of a vaginal stenosis can be made either prepuberty or postpuberty. In prepubertal patients, it can present as hydrocolpos, while post-pubertal presentation includes hematocolpos.

Even if vaginal foreign bodies are uncommon in childhood, their presence and potentially harmful effects on the vagina should be considered whenever a child presents with recurrent pelvic pain and malodorous or bloody vaginal discharge. Furthermore, it is important to consider that the pathological consequences of a foreign vaginal body could be noted after many years. The most common vaginal foreign body includes wadded toilet paper, pencils, hairgrips, safety pins, and batteries.

AA-size alkaline cells and coin lithium button batteries are the most diffuse types of batteries. Alkaline batteries are prone to leak potassium hydroxide, a caustic agent that can cause respiratory, eye, and skin irritation. Batteries have rarely been reported as vaginal foreign bodies. 1 2 3 4 5 In all reported cases, the treatment was based on four combined strategies: removal of the battery, topical and/or systemic antibiotics, intravaginal estrogen cream, and vaginal dilatation.

We reported the staged management of vaginal stenosis following button battery insertion in an 11-year-old girl. To the best of our knowledge, this is the first pediatric case treated with this multimodal approach.

## Case Presentation

An 11-year-old girl experienced at the age of 4 years old a battery insertion in the vaginal canal by her neighbor's son, who was 6-year-old at the time. The girl did not tell her parents about the event immediately, and after approximately 2 weeks, the parents noted the foreign body spontaneously discharged. The girl had not complained of any symptoms at that time and had been asymptomatic for many years after the event.

When she was 11-year-old, she came to our emergency department complaining of cramping pain localized in the lower abdominal region. Additionally, she had mucopurulent discharge not accompanied by fever or systemic symptoms. She underwent an abdominal ultrasound, which showed the presence of hematometrocolpos/pyocolpos. The following day, a vaginal stenosis dilation under general anesthesia was performed. The stenosis was approximately 2 cm proximally to the hymen and had the form of a vaginal diaphragm. Progressive dilation from Hegar number 4 to 22 was performed, evacuating approximately 100 mL of purulent secretions. After the procedure, a 24ch open rubber drain was left in place transvaginally for stenting purposes.

Broad-spectrum intravenous antibiotic therapy (meropenem 20 mg/kg administered three times per day) was given for a week based on the result of the intraoperative swab. The drain was removed at the end of the therapy, and the patient was discharged home. Daily manual dilation with Amielle vaginal dilators was proposed since they are user-friendly, internationally diffused, silicone dilators; however, the parents refused.

After 3 weeks, a vaginoscopy with the patient sedated was conducted. At that stage, the stenosis was still present, not allowing the passage of a Hegar number 4. Furthermore, the girl complained again about recurring abdominal pain during the medical evaluation before the procedure.

A vaginal swab was obtained during the vaginoscopy, and the swab was positive for multiresistance bacteria. As a result, the patient was hospitalized to repeat broad-spectrum intravenous antibiotics for 1 week (meropenem).

**Fig. 1:**
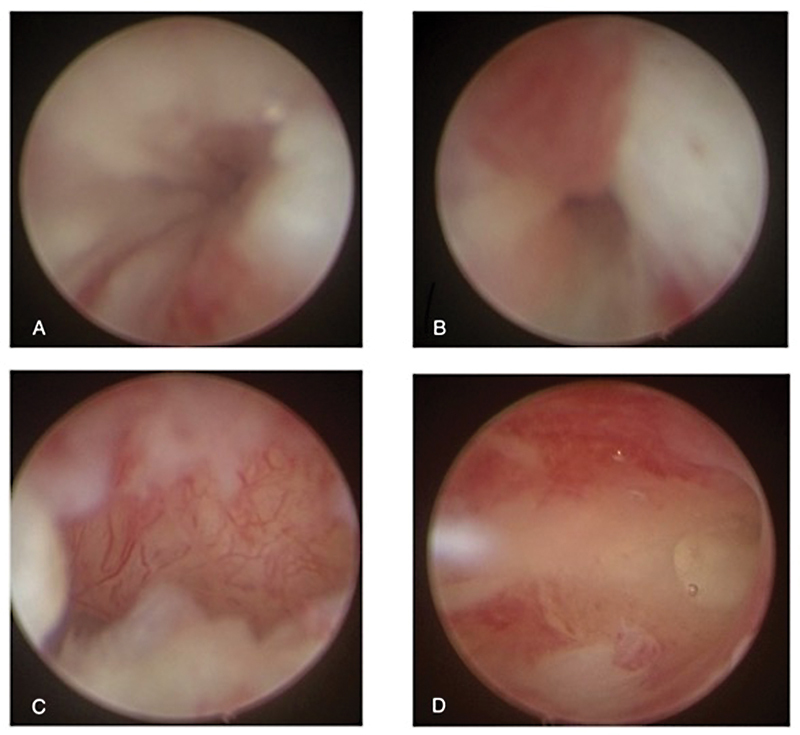
( **A** and **B** ) Vaginoscopic appearance of the stenosis: preincision appearance and ( **C** and **D** ) postincisional aspect.

**Fig. 2:**
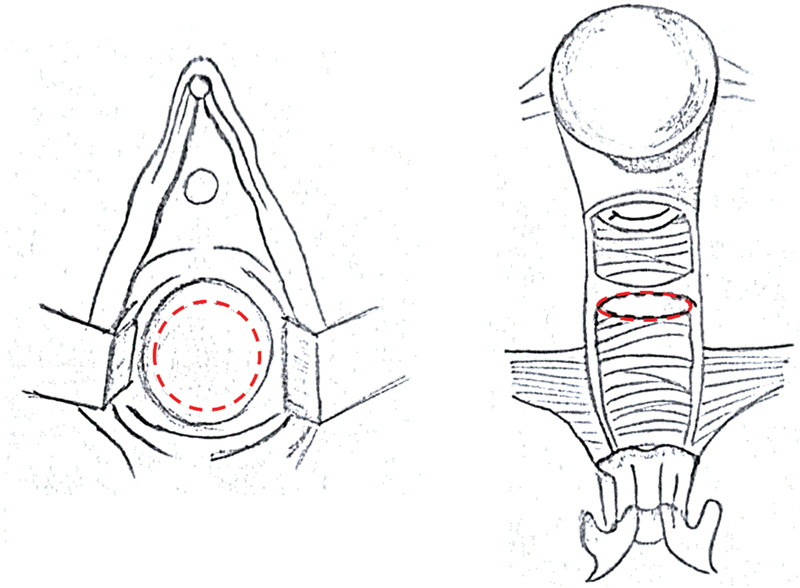
Drawings of the hysteroscopic resection: the red circle indicates the endoscopic incision line.

**Fig. 3:**
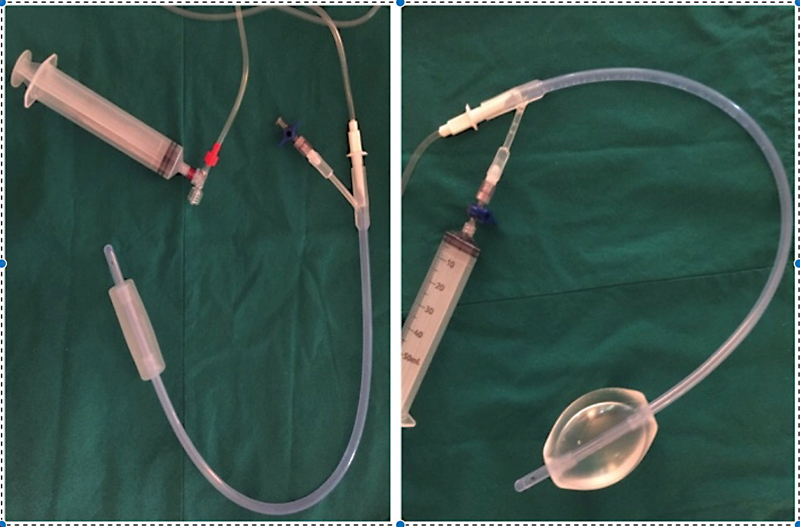
Bakri catheter before and after being filled with water.

After completing her antibiotics therapy, the girl was brought back to the operative room. A vaginoscopic stenosis resection was performed utilizing a monopolar hook through an operative channel ( Figs. 1 and 2 ). The circular stenosis was removed entirely via a circular endoscopic incision, and at the end of the procedure, there was no residual appreciable fibrous ring. A 24 French Bakri catheter filled with 120 mL of distilled water, and a bladder catheter were left in place ( Fig. 3 ). During the procedure, the Bakri catheter was covered with an estrogenic cream to encourage tissue tropism and prevent restenosis. After 10 days, the bladder catheter was removed. The patient was discharged home with the Bakri catheter filled with 100 mL of water. Additionally, the patient was prescribed an estrogenic cream to be applied topically three times a week. After 2 weeks, she was reevaluated in the outpatient setting, where the Bakri was removed. The patient presented with no signs of residual stenosis. A 1-month abdominal ultrasound follow-up was conducted, and the patient was found to have no signs of hematometrocolpos and presented with regular menses. At a 4-month follow-up visit, a bedside ultrasound and digital exploration were performed, which presented with normal findings.

## Discussion

Acquired vaginal strictures are challenging for pediatric surgeons because they are rarely encountered. 6 Acquired stenosis after battery insertion is always related to chemical burn and scarred tissue formation, which is entirely different from congenital stenosis as TVS. TVS can be an isolated malformation caused by a defect of the urogenital sinus and Mullerian conductors in the lower part of the vagina. It can also be a part of the obstructed hemivagina and ipsilateral renal agenesis (OHVIRA) syndrome. 7

Batteries have rarely been reported as vaginal foreign bodies. 1 2 3 4 5 In all the reported cases, the treatment was based on four combined strategies: removal of the battery, topical and/or systemic antibiotics, intravaginal estrogen cream, and vaginal dilatation.

Hegar dilation could be effective if the acquired stenosis is recent. Otherwise, it is only useful for immediately solving the hematometrocolpos and is less effective in the long-term treatment of consolidated stenosis. These cases are more difficult to manage in children compared with adults from both a psychological and an interventional perspective.

As for the pure surgical options, Vassallo and Karram prospectively assessed the outcomes of four approaches to the surgical management of iatrogenic vaginal constriction. 8 Four surgical procedures were considered: Z-plasty, vaginal incision of constriction ring, vaginal advancement, or placement of free skin graft. All patients had been followed up postoperatively, including an assessment of postoperative vaginal caliber. Three patients underwent Z-plasty, eight had incision of vaginal ring or ridge, eight had vaginal advancement, and one underwent placement of a free skin graft. Various clinical criteria were considered in selecting the appropriate corrective operation. Z-plasty was commonly used for midvaginal and introital constrictions when the amount of scar tissue was not too significant. Incision of the ring was employed in more concentric constrictions with extensive fibrosis. Limitations to vaginal advancement primarily included the operator's ability to mobilize adequate healthy vaginal tissue, exposure, and length of the vagina. Free skin grafts had been used in the case of poor mucosal conditions or when excision of the constricting portion left a significant defect.

The mean follow-up was 17 months (range: 3–32 months). Vassallo and Karram concluded that the appropriate surgical procedure depends on the site and extent of the vaginal constriction, the state of the surrounding tissue, and the overall length and caliber of the vagina ( Table 1 ).

**Table 1:** Different techniques used for acquired vaginal stenosis

Author	Surgical technique	Number of pts	Results
Vassallo and Karram	• Z-plasty • Incision of constriction ring • Vaginal advancement • Placement of free skin graft	20 cases	17-mo FU: 75% uneventful; 25% needed a second procedure
Layman and McDonough	Olbert balloon catheter	6 cases	Uneventful FU
Cheng et al	Vaginoscopic management using a “no-touch” technique with a diagnostic 4.5-mm-outer sheath hysteroscope	14 cases	1-y uneventful FU
Kamal et al	Surgical resection and postoperative vaginal dilation	1 case	2-y uneventful FU

The surgical management of an obstructive TVS with a proximal hematocolpos can be demanding since a safe entry is required to approach the obstructed vagina from below. This approach includes pulling the redundant vaginal mucosa caudally in the direction of the operator, which would take advantage of the excessive vaginal tissue, as in the present case. Layman and McDonough proposed the use of the Olbert balloon catheter to facilitate this kind of surgical management. 9 The Olbert balloon catheter is an inflatable balloon catheter with radiopaque markers. Once expanded, it can be pulled caudally in the direction of the vaginal outlet to optimize the exposure of the vaginal septum, facilitating the surgical incision. Through this method, the group was able to treat six cases successfully.

In 2019, Cheng C et al proposed a valid alternative to vaginoplasty. While the group treated a tissue different from the scarred tissue of acquired stenosis, this modality may constitute an option in selected cases. This method was performed under direct visualization using a speculum or vaginal retractors for stenosis in virgin adolescents with the OHVIRA syndrome. Based on vaginoscopic management and using a “no-touch” technique, a diagnostic 4.5-mm-outer sheath hysteroscope was inserted into the vagina cautiously through the hymenal ring. 7 Under ultrasound guidance, an 8-mm outer sheath unipolar or bipolar resecto-hysteroscope fitted with an L-hook electrode was then introduced into the vagina. Next, a longitudinal incision was made at the prominent bulge of the vaginal septum, subsequently extended cranially and caudally. A 16 Fr Foley catheter was sequentially inserted between the incised halves of the septum, and the balloon was inflated with up to 80 mL of air and left in place for 2 days. The 1-year follow-up was uneventful.

Kamal et al underlined that vaginal restenosis at the resection site remains the most common surgical complication. To avoid this, they proposed routinely postoperative vaginal dilation. 10

In this case, the use of the Bakri catheter in the second attempt to solve the stenosis was an innovative step and required cooperation with the adult gynecologist. Bakri catheters are usually indicated to stop postpartum hemorrhages. Compared with the Foley catheter, the Bakri catheter has a longer balloon (approximately 10 cm), more stability, and is not prone to dislodgment. In our patient, the catheter was filled with distilled water and covered with estrogenic cream to impart a mechanical expansion. The device was well tolerated by the patient, and we were able to discharge her with this catheter in place, allowing for a safer stabilization of the endoscopic resection.

Fistula formation is a well-known complication from battery ingestion, including the life-threatening acquired aortoesophageal fistula. In the literature, no report currently exists regarding fistula formation after battery vaginal insertion, but it is biologically plausible to have similar complications in the vagina. 11

Clinicians should be aware and consider the possible complication of prolonged battery placement, especially in prepubertal girls with less estrogenized vaginal mucosas. A more estrogenized vaginal environment allows the increasing permeation through the vaginal tissue, blood permeability, and general tissue tropism, avoiding tissue ischemia, necrosis, and possible fistula formation. Tissue manipulation and dissection should be done carefully when incising the stenotic part, avoiding lesions to the bladder or bowel that may result in a vesicovaginal or rectovaginal fistula. Warning clinical signs could be passage of gas, stool, pus or urine leaks from the vagina, foul-smelling vaginal discharge, or recurrent vaginal or urinary tract infections. Nevertheless, data exist, most of all in adult patients, about the long-term effects of prolonged battery retention including scarring, dyspareunia, sexual dysfunction, late recurrence, or intermenstrual bleeding.

## Conclusions

Acquired vaginal stenosis can be demanding to treat, particularly with the sole conservative approach. After considering the consistency and the extent of the stenosis, the Hegar dilation can be a first-line option. In the pediatric population, vaginal dilators may not be a preferred treatment method by patients and their parents. The endoscopic approach can be a second-line, minimally invasive treatment however, long-term outcomes are difficult to predict. A multidisciplinary approach involving adult gynecologists is the optimal way to manage these rare cases.

## Notes

New Insights and Importance for a Pediatric Surgeon Acquired vaginal strictures are challenging for pediatric surgeons because they are rarely encountered. Acquired stenosis after battery insertion is always related to chemical burn and scarred tissue formation, which is entirely different from congenital stenosis. Acquired stenosis could be demanding to treat, particularly with the sole conservative approach. A first-line option can be the Hegar dilation. The endoscopic approach can be a second-line, minimally invasive treatment, but longterm outcomes are difficult to predict.
